# Characterization of proliferating cell nuclear antigen (PCNA) from pathogenic yeast *Candida albicans* and its functional analyses in *S. Cerevisiae*

**DOI:** 10.1186/s12866-015-0582-6

**Published:** 2015-11-04

**Authors:** Kodavati Manohar, Narottam Acharya

**Affiliations:** Laboratory of Genomic Instability and Diseases, Department of Infectious Disease Biology, Institute of Life Sciences, Bhubaneswar, 751023 India

**Keywords:** Sliding clamp, Native PAGE, Complementation, Hydroxyurea, DNA damage tolerance, Surface plasmon resonance

## Abstract

**Background:**

Proliferating cell nuclear antigen (PCNA/POL30) an essential protein forms a homotrimeric ring encircling dsDNA and serves as a molecular scaffold to recruit various factors during DNA replication, repair and recombination. According to Candida Genome Database (CGD), orf19.4616 sequence is predicted to encode *C. albicans* PCNA (CaPCNA) that has not been characterized yet.

**Results:**

Molecular modeling studies of orf19.4616 using *S. cerevisiae* PCNA sequence (ScPCNA) as a template, and its subsequent biochemical characterizations suggest that like other eukaryotic PCNAs, orf19.4616 encodes for a conventional homotrimeric sliding clamp. Further we showed by surface plasmon resonance that CaPCNA physically interacted with yeast DNA polymerase eta. Plasmid segregation in genomic knock out yeast strains showed that CaPCNA but not its G178S mutant complemented for cell survival. Unexpectedly, heterologous expression of CaPCNA in *S. cerevisiae* exhibited slow growth phenotypes, sensitivity to cold and elevated temperatures; and showed enhanced sensitivity to hydroxyurea and various DNA damaging agents in comparison to strain bearing ScPCNA. Interestingly, wild type strains of *C. albicans* showed remarkable tolerance to DNA damaging agents when compared with similarly treated yeast cells.

**Conclusions:**

Despite structural and physiochemical similarities; we have demonstrated that there are distinct functional differences between ScPCNA and CaPCNA, and probably the ways both the strains maintain their genomic stability. We propose that the growth of pathogenic *C. albicans* which is evolved to tolerate DNA damages could be controlled effectively by targeting this unique fungal PCNA.

**Electronic supplementary material:**

The online version of this article (doi:10.1186/s12866-015-0582-6) contains supplementary material, which is available to authorized users.

## Background

Proliferating cellular nuclear antigen (PCNA), an evolutionarily conserved protein found in eukaryotes and archaebacteria serves as a docking platform for many proteins that function in DNA replication, repair, recombination, cell cycle, and chromatin remodeling [1, 2]. It belongs to a family of sliding clamps that increase processivity of replicative DNA polymerases [3, 4]. Despite little sequence similarity among the sliding clamps in all domains of life, they possess superimposable three-dimensional structures with highly conserved functions [1]. A typical sliding clamp comprises of multimeric, toroidal-shaped structures with pseudo-six fold symmetry that encircles the DNA double helix [5, 6]. While β-clamp, the sliding clamp of eubacteria is a homodimer; PCNA of eukaryotes and T4 bacteriophage is a homotrimer, and a heterotrimer in archaea [7, 8]. Each PCNA monomer consists of two globular topologically identical domains connected by an inter domain connecting loop (IDCL). The three dimensional structure reveals that the inner surface of the PCNA ring is comprised of 12 α-helices with suitably exposed positively charged amino acids to interact with the sugar-phosphate backbone of DNA, whereas the outer surface is composed of 54 β-sheets and three IDCLs appropriate for protein–protein interactions [5, 6]. PCNA by itself cannot directly encircle DNA; rather it is loaded by a eukaryotic clamp loader, RFC [9]. The loaded PCNA ring only recruits polymerases and other factors firmly to DNA, making the sliding clamp an essential cofactor for DNA transaction processes. Apart from DNA polymerases, PCNA is found to interact with proteins such as FEN1, DNA glycosylases, DNA ligase I, p21, CCK2, cyclin D, DNA methyltransferases etc. [2, 10–16]. PCNA interacts with its partners mostly through its IDCL but occasionally by its C-terminus, the center loop and the backside loop [17] .

Mutational analysis of yeast PCNA has suggested separate roles for PCNA in DNA replication and repairs [18, 19]. For example; PCNA mutants like *pol30-9* (ED104,105AA) and *pol30-22* (DE256,257AA) show sensitivity to DNA methylating agents and UV light, however they grow normally on medium containing a replication inhibitor hydroxyurea, and do not elicit any temperature sensitivity. Similarly *pcna-201* (C22Y) and *pcna-204* (C81R) alleles generate frame-shift and substitution mutations due to reduced MMR efficiency but do not show any defects in replication or other repair processes. *In vitro* binding studies revealed a compromised interaction of these mutant proteins with MSH2 and MSH6 proteins. A number of PCNA mutants have also been characterized those are defective in both DNA replication and repair processes. Alleles like *pcna-79* that has IL126, 128AA mutations in the IDCL, and *pcna-90* that carries PK252, 253AA mutations in the carboxyl terminal tail exhibit growth defects, elevated sensitivity to DNA-damaging agents, and higher rates of spontaneous mutations [17]. Biochemical characterization of some of these mutant PCNAs *e.g.* PCNA-240 often showed structural alteration and reduced stability of the trimeric ring [19].

PCNA has been characterized in human, rat, budding and fission yeasts, protozoan, flies, Arabidopsis, and many archaeal species [7, 20–25]. However, no such studies of PCNA from pathogenic *Candida albicans* or any other *Candida* species have been carried out till date. *C. albicans*, an opportunistic pathogen, which asymptomatically colonizes humans, can cause severe infection in immounocompromised people or when the microbiotic balance is disturbed [26, 27]. Systemic fungal infections by *C. albicans* are life threatening and of great concern to human health [28]. Study of the fundamental cellular processes such as DNA replication, repair and recombination in this pathogenic yeast and their parallel comparison with nonpathogenic *S. cerevisiae* is crucial to unravel the distinct biochemical processes or factors, which may be targeted to control infections/diseases. DNA repair mechanisms like base excision repair (BER) and nucleotide excision repair (NER) are well studied in *C. albicans*; and interestingly unlike in *S. cerevisiae*, DNA repair genes such as NTG1, APN1, OGG1, RAD2 and RAD10 do not to play any crucial roles in mutagenesis, genomic stability and drug resistance phenotype in *C. albicans* [29]. Similarly, proteins involved in mismatch repair (MMR) and non-homologous end joining (NHEJ) also do not contribute significantly to the *Candida* genomic stability; however defects in double-strand break repair enhance genomic instability [30]. Role of DNA replication components in genomic stability have not yet been explored in this pathogenic fungus. Thus, to initiate understanding of DNA replication in this model organism, in this study we report on the biochemical characterization of CaPCNA as well as its role in DNA replication and repair in *S. cerevisiae.*

## Results

### Identification, sequence analysis and structural comparison of CaPCNA with *S. cerevisiae* PCNA

**Fig. 1:**
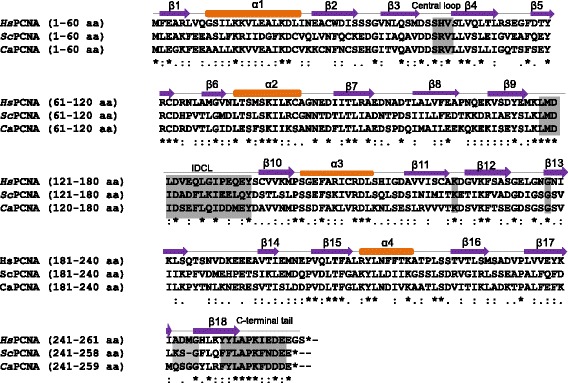
*In silico* analysis of CaPCNA. The amino acid alignment and secondary structure prediction were carried out using CLUSTAL W and PSIPRED v3.3 programs, respectively. Identical residues are marked by *, whereas the similar residues are marked with : symbols. Conserved motifs and residues like central loop, IDCL, C-terminal tail, K164 and G178 were shaded grey. All the theoretically predicted α-helices (cylinder) and β-strands (arrow) of CaPCNA are numbered.

To identify a putative homologue of *S. cerevisiae* PCNA, the amino acid sequence was used for BLAST analysis. BLAST analysis identified a *C. albicans* SC5314 homologue that corresponds to orf19.4616 (alias CaO19.12086) in CGD with a score of 6e^−103^, identity of 54 % and similarity of 81 %. Henceforth we refer to this orf as CaPCNA. Interestingly, CLUSTAL W alignment analysis showed 42 % and 35 % identity of *Candida* and yeast PCNA with that of human, respectively (Fig 1). In addition, the sequence identity among the PCNAs was found throughout the orfs in clusters of three to four amino acids. CaPCNA sequence encodes a predicted 29 kDa protein composed of 259 amino acids which is two amino acids shorter than HsPCNA (28.7 kDa) but one amino acid longer than ScPCNA (28.9 kDa) at its carboxyl terminus. Alignment also showed very high degree of amino acid conservation in the central loop (43SRV45), inter domain connecting loop (IDCL, 118L----D134) as well in the C-terminal tail (149 F---E259); and these domains are known to interact with different protein factors during DNA synthesis. Regulatory residues like Lys164 that undergoes ubiquitylation and sumoylation; and Gly178 which is involved in stability of the PCNA ring are found to be conserved [31, 32].

**Fig. 2:**
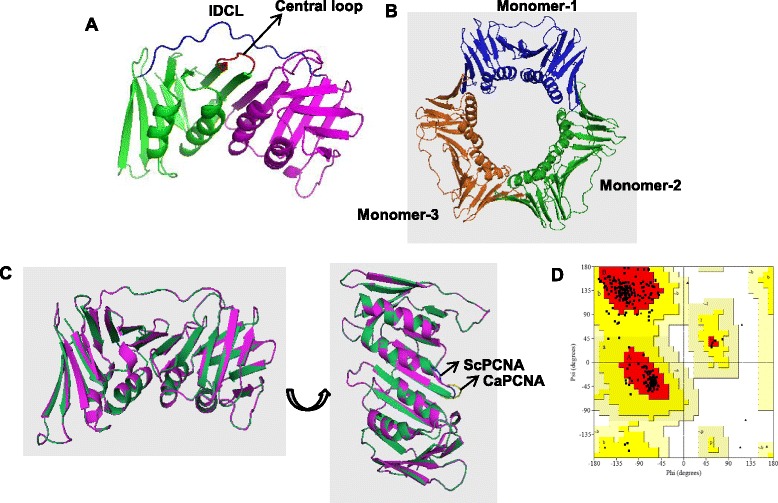
Model structure of CaPCNA*.* **a**. Structure of CaPCNA monomer showing N- terminal domain in green, C-terminal domain in purple and IDCL in blue. **b**. Ring structure of CaPCNA depicting three monomers connecting N-terminal domain with C-terminal domain. **c**. Superimposed structures of CaPCNA (purple) and ScPCNA (green); and non-overlapping portions of CaPCNA (yellow) and ScPCNA (blue) are shown. **d**. The Ramachandran plot of the homology-modeled structure of CaPCNA. The red regions indicate “most favored”, yellow colored areas “additional allowed” and white portion represent “disallowed”.

Since the sliding clamps from all domains of life possess superimposable three-dimensional structures and primary sequence of CaPCNA showed remarkable similarity, we examined the structure of putative CaPCNA by using *in silico* approach. By using SWISS MODEL and taking ScPCNA pdb structures as template, model structure of CaPCNA was predicted (Fig. 2). CaPCNA also forms a toroidal shaped homotrimeric ring, and the superimposition of the modeled complex with the template ScPCNA showed a root mean square deviation (RMSD) of 0.48 Å (Fig. 2 B and C). The Ramachandran plot showed 88.2 % residues in the most favoured and 11.8 % residues in the additional allowed region (Fig. 2 D) confirming the acceptability of the model structure. In the monomeric structure, CaPCNA is composed of two identical globular domains, one with β1-α1-β2-β3-β4-β5-β6-α2-β7-β8-β9 and the other with β10-α3-β11-β12-β13-β14-β15-α4-β16-β17-β18, joined by an IDCL (in blue, Fig. 2 A). The minor difference in CaPCNA model structure was noticed in the loop that connects β17 and β18 at the C-terminal tail (Fig. 2 C).

### CaPCNA forms a stable homotrimeric ring

**Fig. 3:**
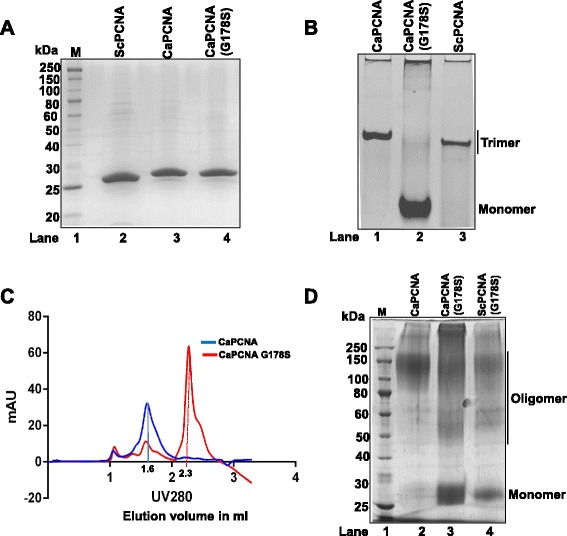
Purification and biochemical analysis of PCNAs. The wild type and mutant PCNAs were purified and ~1 μg of each protein was analyzed. **a** *.* Purified proteins were resolved in SDS-12 % polyacrylamide gel. Lanes 1, Molecular weight standards; 2, ScPCNA; 3, CaPCNA; and 4, CaPCNA G178S. **c**. Purified proteins were resolved in 12 % non-denaturing polyacrylamide gel. Lanes 1, CaPCNA; 2, CaPCNA G178S; and 3. ScPCNA. **c.** Size exclusion chromatograms of wild type (blue) and G178S mutant (red) CaPCNAs with the peak fractionation at ~1.6 ml and ~2.3 ml elution volume, respectively, were shown. **d**. Glutaraldehyde crosslinked 20 μg of CaPCNA (lane 2) or CaPCNA G178S (lane 3) or ScPCNA G178S (lane 4) proteins were resolved in SDS-12 % polyacrylamide gel. Monomer (~29 kDa) and oligomeric PCNAs (~60-180 kDa) are marked. Lane 1, Molecular weight standards.

**Fig. 4:**
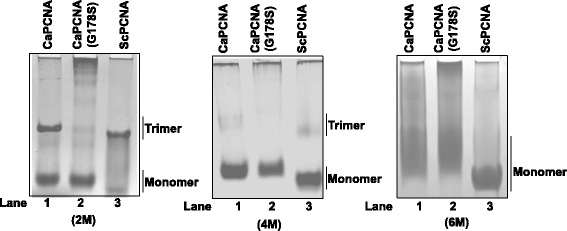
Stability of PCNAs. Purified proteins were resolved in 12 % polyacrylamide gel containing 2 M, 4 M, and 6 M urea as indicated, and stained by Coomassie Brilliant Blue R250. Lanes 1, CaPCNA; 2, CaPCNA G178S; and 3. ScPCNA.

To characterize CaPCNA further, the orf was PCR amplified and expressed in bacterial cells. The orf was in frame with N-terminal GST and a Prescision protease site was strategically placed to cleave PCNA from GST. Using glutathione sepharose beads as described in methods, wild type and mutant PCNA were purified to near homogeneity. As predicted, CaPCNA (29 kDa, pI 4.54) and its G178S mutant migrated relatively slower than ScPCNA (28 kDa, pI 4.42) when analyzed by SDS-PAGE (Fig. 3 A, compare lane 2 with 3 & 4). Structural modeling of CaPCNA sequence predicted it to form an oligomer of three subunits, to authenticate the prediction the purified proteins were analyzed by native PAGE. Expectedly, CaPCNA migrated at a position similar to ScPCNA. As the latter is a homotrimer, this observation suggested that CaPCNA also forms a stable trimer (Fig. 3 B, lane 1 & 3). A slightly faster mobility of ScPCNA may be attributed to its lower pI comparison to CaPCNA. Biochemical and structural characterization of ScPCNA G178S mutant suggested that G178 residue resides in the interface of the two monomers and due to mutation to serine, it destabilizes the PCNA ring; therefore it remains mostly as monomer [33]. To ascertain co-migration of wild type CaPCNA with ScPCNA in native PAGE is because of oligomerisation of monomers and not due to any abnormality in CaPCNA, a G178S mutant protein was further analyzed. As reported earlier, CaPCNA G178S mutant (Fig. 3 B, lane 2) migrated faster than the wild type *Candida* and yeast PCNA, probably as monomer and suggesting an evolutionary conservation of its role in stabilization of PCNA ring. Size exclusion chromatography (Fig. 3 C) confirmed the trimeric nature of the protein as wild type CaPCNA and ScPCNA eluted around ~90 kDa (~1.6 ml elution volume, blue line). However candida G178S mutant PCNA eluted much later as ~30 kDa (~2.3 ml elution volume, red line) when referred to the molecular weight standards (Fig. 3 C). Further we verified the stability of the PCNA trimers by resolving them on a urea containing PAGE (Fig. 4). Both *Candida* and budding yeast PCNA showed maximum stability in 2 M urea and complete urea dependent denaturation to monomer at 6 M urea. Here the CaPCNA G178S mutant protein served as a marker of monomeric form of PCNA (Fig. 4 lane 2).

Although all eukaryotic PCNAs characterized so far form a stable trimer *in vitro*, the exact composition of PCNA ring in the cell is still not clear as formaldehyde cross-linked CHO cells extract or purified human PCNA resulted in formation of a double trimeric PCNA [34]. Mutational analysis suggested the role of R5 and K110 amino acids of human PCNA in doublet-trimer formation. Reagents like formaldehyde or glutaraldehyde cross-links neighboring lysine or arginine residues of monomers by amine bonds to form oligomers. As these residues (K5 and K110) are also conserved in CaPCNA, purified recombinant proteins were cross-linked and analyzed on 12 % SDS-PAGE (Fig. 3D). Like HsPCNA, CaPCNA showed a smear ranging from ~90-180 kDa (trimer and double trimer) whereas G178S PCNA mutant migrated in multiple forms by glutaraldehyde cross-linking and predominantly as monomers (29 kDa). Mutant PCNA also formed dimeric PCNA (~60 kDa). Similar results are also obtained with ScPCNA and its G178S mutant (Fig. 3D lane 4). In consistent with earlier findings, our study also suggests that PCNA from *C. albicans* and *S. cerevisiae* may also exist as a double-trimer in the cell.

### CaPCNA physically interacts with yeast DNA polymerase eta (ScPolη)

**Fig. 5:**
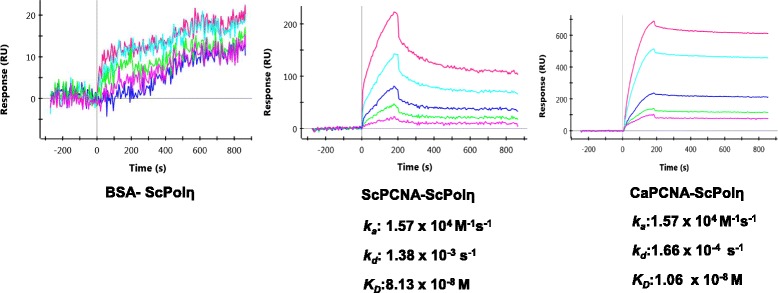
Physical interaction of CaPCNA with ScPolη: About 25-500nM of ScPolη was injected on CaPCNA or ScPCNA or BSA immobilized GLC chip as indicated with running buffer at a flow rate of 50 μl/min for 180 s with a 600 s dissociation phase. The dissociation constants were determined.

PCNA functions as a docking site for DNA polymerases and other proteins during various processes of DNA transaction. In order to function, CaPCNA needs to interact with DNA polymerases and other associated proteins. Yeast DNA polymerase eta (ScPolη) that takes part in translesion DNA synthesis [35] was used as a candidate polymerase for physical interaction study by surface plasmon resonance. We immobilized CaPCNA or ScPCNA on a GLC- chip to determine the binding affinity for ScPolη. As a control, required amount of BSA was also immobilized. When ScPolη was passed over BSA, we did not see any significant increase in response unit where as it showed ~250 and ~650 RU for ScPCNA and CaPCNA, as ligands respectively (Fig. 5) suggesting the tight binding between PCNAs and DNA polymerase. The rate of dissociation of ScPolη from ScPCNA surface (*kd*:1.38 x 10^−3^ s-1) was comparable to that from CaPCNA immobilized surface (*kd*:1.66 x 10^−4^ s-1). The *K*_*D*_ values of CaPCNA and ScPCNA for ScPolη were determined as 10.6 nM and 80.13 nM, respectively.

### CaPCNA complements essential function of POL30 in S. cerevisiae

**Fig. 6:**
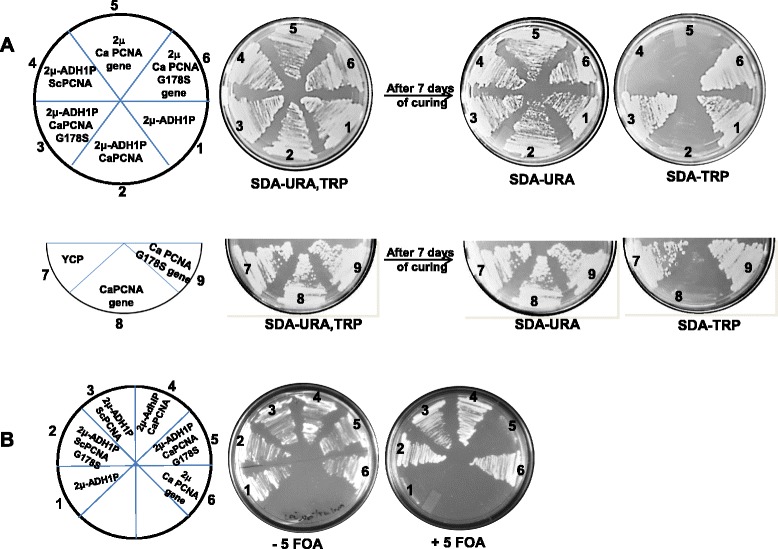
Complementation analysis of PCNAs: **a**. YTS9 strains (trp+) containing various plasmids were selected on synthetic media without tryptophan and uracil. These transformants were grown only on liquid media omitting uracil at 30 °C to cure the resident plasmid as described in methods. After seven such consecutive sub-culturing, presence of the nutritional markers were tested by plating on SD agar plates lacing uracil alone, or tryptophan alone. Plates were incubated for 2 days at 30 °C and photographed. Sectors 1 & 7, empty vectors; 2, 2 μ, ADH1p-CaPCNA, URA3; 3, 2 μ, ADH1p-CaPCNA G178S, URA3; 4, 2 μ, ADH1p-ScPCNA, URA3; 5, 2 μ, CaPCNA gene, URA3; 6, 2 μ, CaPCNA gene G178S, URA3; 8, CEN, CaPCNAgene, URA3; and 9, CEN, CaPCNAgene G178S, URA3. **b**. The transformants of yeast strain YNA05 (ura+) containing various plasmids were selected on SD media w/o leucine and uracil. Isolated colonies were picked and re-streaked on SD-leucine plate but with or without 5-FOA. Sectors 1, empty vector; 2, 2 μ, ADH1p-ScPCNA G178S, LEU2; 3, 2 μ, ADH1p-ScPCNA, LEU2; 4, 2 μ, ADH1p-CaPCNA, LEU2; 5, 2 μ, CaPCNA G178S, LEU2; 6, 2 μ,CaPCNA gene, LEU2.

*POL30* gene in any organism is indispensable for cell survival. Because our biochemical and structural modelling studies suggested CaPCNA to be alike of ScPCNA, it compelled us to examine whether CaPCNA can perform the essential functions of yeast *POL30* and support cell viability of the genomic null strain. To achieve our goal CaPCNA was expressed either under its own promoter or under a yeast constitutive promoter *ADH1* in genomic deletion strains of *S. cerevisiae* but survives due to presence of a plasmid bearing ScPCNA. The segregation of plasmids was carried out to replace the ScPCNA with CaPCNA by two different approaches. In one approach, YTS9 strain which is null for genomic *POL30* but carrying YCP-*ScPOL30 DE41, 42AA*-TRP1 plasmid for survival was used to obtain transformants harbouring various CaPCNA plasmids (URA3) on SD media lacking tryptophan and uracil. Attempt to cure the retained plasmid was carried out by repeated sub-culturing of the transformants on SD-uracil liquid media as described in methods. About 30 isolated colonies from each set were streaked on SD-uracil and SD-tryptophan plates. The percentage of curing of resident plasmid was scored and a representative figure has been shown (Fig. 6 A). The lack of growth on SD-tryptophan plate but growth on SD-uracil will suggest complete curing of the resident PCNA and complementation due to incoming plasmid. Strains containing CaPCNA or its gene in both CEN/2 μ vectors were able to replace the resident plasmid (Fig. 6 A sectors 2, 5 and 8), and about 100 % efficient curing was achievable. Similar result was also obtained for incoming ScPCNA but not for the vector controls as both the nutritional marker phenotypes (URA and TRP) were retained (Fig. 6 A compare sector 4 with 1 and 7).

In the other approach various CaPCNA constructs were transformed into a yeast strain YNA05 in which the chromosomal *POL30* gene was deleted and the cell viability was maintained by ADH1p-ScPCNA-URA3 based plasmid to allow positive as well as counter selection of this plasmid by 5-FOA. As shown in Fig. 6B, cells harboring CaPCNA or ScPCNA but not the vector control could grow on 5-FOA containing SD medium, in which the resident plasmid (URA3) was negatively selected (compare lane 1 with 3, 4 and 6). Taken together, we conclude that CaPCNA is able to functionally complement the essential functions of yeast *POL30* and supports cell viability.

### CaPCNA G178S mutant functions differently in yeast

The *pol30-178,* also known as *re*v*6-1 S. cerevisiae* allele that carries a genomic mutation of G178 to serine in PCNA is well characterized [32]. Biochemical characterizations from our study as well as from those of other’s suggested the crucial role of this residue in stabilizing the PCNA ring as both *Candida* and yeast G178S mutant proteins remained mostly as monomers *in vitro*. Although *pol30-178* allele strain grows normally, surprisingly, *Candida* G178S did not complement the essential function of *ScPOL30* when plasmid segregation was carried out in both YTS9 and YNA05 strains (Fig. 6A sectors 3, 6 and 9). However, the incoming ScPCNA G178S was able to complement (Fig. 6B, compare sector 2 with 5) the cell growth on FOA plate. Our observations suggest that presumably ScPCNA G178S is able to form a DNA clamp *in vivo*, whereas *Candida* mutant is unable to form a functional clamp in yeast.

### CaPCNA exhibits slow growth phenotype in yeast and partially complements replication defects caused by hydroxyurea

**Fig. 7:**
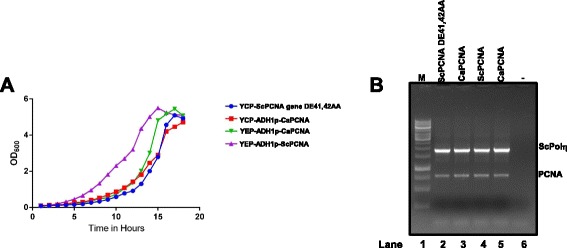
Growth curves of various yeast strains expressing CaPCNA: **a**. Genomic *POL30* deletion yeast strains containing YEP-ADH1p-ScPCNA (−−▲--), YCP-ADH1p-CaPCNA (−−■--) , YEP-ADH1p-CaPCNA (−−▼--), and YCP-ScPCNA gene with DE41,42AA (--●--) were grown in 100 ml YPD liquid medium at 30 °C and absorbance at 600 nm were measured at regular intervals. The plot was an average three sets of experiment. **b.** Total RNA from the above strains were isolated and semi-quantitive Reverse Trascriptase PCR was carried out. The PCR products were analyzed in a 1 % agarose gel electrophoresis. Lane 1, 1 KB DNA ladder; Lane 2 c-DNAs of ScPCNA DE41,42AA and ScPolη; Lane 3 c-DNAs of CaPCNA and ScPolη; Lane 4 c-DNAs of ScPCNA and ScPolη; Lane 5 c-DNAs of CaPCNA and ScPolη; Lane 6 no c-DNA control reaction.

To analyze the role of CaPCNA in DNA replication, growth rates of *POL30* knock out yeast strains expressing CaPCNA in single to multi-copy plasmids were monitored. Interestingly, strains harbouring CaPCNA formed smaller sized colonies and grew relatively slower than that with wild type ScPCNA, and accordingly, strain having CaPCNA in a CEN plasmid (low copy number) grew even slower (Fig. 7A, see Additional file 1: Table S1). As previously reported, we also observed a slow growth phenotype for YTS9 strain; and the slow growth phenotype was attributed to the weak binding of mutant PCNA (DE41, 42AA) with DNA and DNA polymerases [18]. Similarly, yeast strain expressing CaPCNA or ScPCNA DE41, 42AA showed temperature sensitivity at 14 °C and 35 °C (data not shown). To ascertain the slow growth defects of various yeast strains is not due to the difference in the expression levels of PCNAs rather due to the intrinsic properties of CaPCNA, RT-PCR was carried out to check the mRNA level of both the PCNA. Our result showed that there is no noticeable difference in the expression between the two PCNAs under *ADH1* or under its own promoter (Fig. 7B, compare lanes 2, 4 with 3, 5). The native expression level of yeast DNA polymerase eta was checked and used as a loading control. The relative fold change of band intensities of PCNA vs ScPolη varies from ~ 0.7 to 0.9 (see Additional file 2: Table S2).

**Fig. 8:**
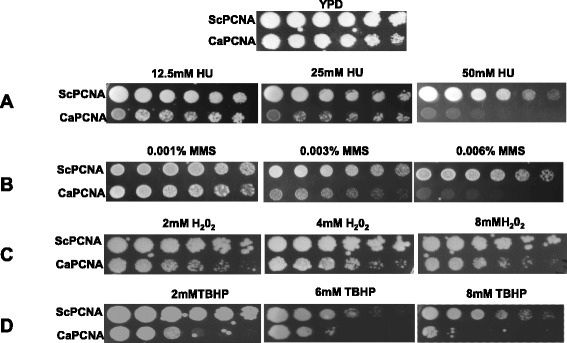
Sensitivities of *S. cerevisiae* strains to HU and DNA damaging agents. Cells of genomic *POL30* deletion yeast strains containing YEP-ADH1p-ScPCNA or YEP-ADH1p-CaPCNA from an overnight YPD culture were serially diluted and spotted onto YPD plates without or with indicated concentrations of HU, MMS, H_2_O_2_ and TBHP. The plates were incubated at 30 °C for 3 days and then photographed.

Hydroxyurea depletes cellular dNTPs level and has been widely used as a replication inhibitor. When we examined susceptibility of these strains to HU (Fig. 8A), yeast strain ectopically expressing ScPCNA (YEP-ADH1p-ScPCNA) did not show any significant sensitivity up to 50 mM HU, rather surprisingly strain expressing CaPCNA (YEP-ADH1p-CaPCNA) showed hypersensitivity to similar concentration of HU, as growth was quite adversely affected on YPD plates containing 50 mM HU. However growth at 12.5 mM HU for both the strains remained unaffected. In this assay also both the strains displayed differential growth rates and yeast strain expressing homologous PCNA was considerably over-growing the other. Although CaPCNA complemented essential nature of Sc*POL30* and was able to support DNA replication, it was evident from the growth curve, temperature and HU sensitivity analyses that CaPCNA is not as efficient as ScPCNA as far as DNA replication is concerned in *S. cerevisiae*.

### Ectopic expression of CaPCNA caused sensitivity to DNA damaging agents in yeast

Eukaryotic PCNA functions in DNA repair in addition to its role in DNA replication. In order to study the function of CaPCNA in DNA repair, strains were subjected to various damaging agents like MMS, H_2_O_2_ and TBHP (Fig. 8B-D). Like treatment with HU, strains expressing CaPCNA displayed significant sensitivity to these agents than that containing ScPCNA in yeast. MMS an alkylating agent methylates DNA mainly to generate adducts like N7-methylguanine and N3-methyl adenine; and is highly mutagenic. About 0.006 % MMS is highly toxic to yeast bearing CaPCNA but strain having ScPCNA showed negligible sensitivity (Fig. 8 B). H_2_O_2_ and TBHP increases oxidative stress in a cell and generate oxidized DNA lesions. At 8 mM of these reagents, growth of yeast bearing CaPCNA but not of strain having ScPCNA was impaired (Fig. 8C and D). Taken together, we conclude that CaPCNA is relatively less efficient in coordinating DNA repair processes in yeast.

### *C. albicans* is genetically resistant to DNA damaging agents in comparison to nonpathogenic *S. cerevisiae*

**Fig. 9:**
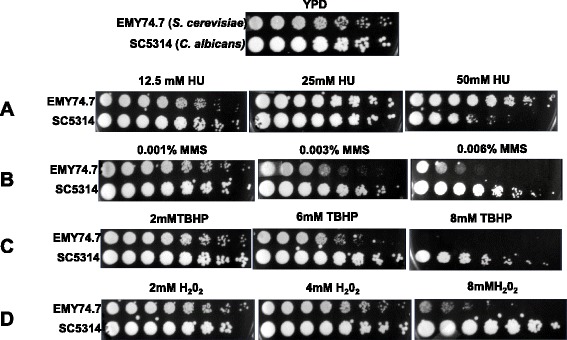
Sensitivities of wild type *C. albicans SC5314* and *S. cerevisiae EMY74.7* to HU and DNA damaging agents. Cells from an overnight YPD + uridine culture were serially diluted and spotted onto YPD + uridine plates without or with indicated concentrations of HU, MMS, H_2_O_2_ and TBHP. The plates were incubated at 30 °C for 3 days and then photographed.

Heterologous expression of CaPCNA in non-pathogenic *S. cerevisiae* resulted in sensitivity to DNA damaging agents, however during infection *C. albicans* evades various attacks of ROS/RNI species generated by innate immune cells [36]. Whether in a homologous system CaPCNA provides any protective role, a comparative study of susceptibility by wild type strains of *C. albicans* and yeast to DNA damaging agents was carried out (Fig. 9). When equal number cells were diluted and spotted; interestingly, *C. albicans* strain SC5314 which is a commonly used laboratory wild type strain overgrew *S. cerevisiae* strain EMY74.7, a derivative of widely used DBY747. So the retardation of growth rate in Fig. 7A where we checked growth rates of yeast containing CaPCNA is not due to the characteristic CaPCNA rather because of lack of species specific interaction with replication components. Despite the differences in the growth rate, *Candida* SC5314 exhibited moderate sensitivity to HU than yeast EMY74.7 at 50 mM HU, the reason for such a phenotype requires further extensive analysis (Fig. 9 A). The *C. albicans* SC5314 showed tolerance to DNA damaging agents like MMS, H_2_O_2_, and TBHP unlike yeast strain expressing CaPCNA; and at same or lower concentration of reagents, a significant impairment of growth of EMY74.7 was observed. In a different study, similar results have been reported earlier by using diploid strains of *S. cerevisiae* BY4743 and *C. albicans* DKCa39 [29, 30]. While one copy of PCNA gene can be easily deleted from diploid *S. cerevisiae*, because of haplo-insufficiency similar deletion was not achievable in *C. albicans* [37, 38]. Taken all together we suggest that the fungal pathogen *C. albicans* is genetically evolved to be resistant to DNA damages in comparison to nonpathogenic budding yeast; and CaPCNA dependent DNA damage tolerance pathways could play a crucial role in addition to other cellular mechanisms like efficient drug efflux pumps, thicker cell wall and any other components present in *C. albicans* to maintain genomic stability.

## Discussion

Systemic infections caused by *Candida albicans* are life threatening in immune-compromised individuals [28]. In order to kill the microbes, the innate immune cells like macrophages phagocytize *Candida* cells, generate ROS/RNI that damage DNA [26, 36]. Therefore *C. albicans* becomes an interesting model system to understand mechanisms of DNA transaction processes and their parallel comparison with well-studied nonpathogenic *S. cerevisiae* is crucial to unravel the distinct biochemical processes or factors, which may be targeted to control infections/diseases. For maintenance of genomic stability and cell survival, PCNA plays an essential role and regulate recruiting of factors such as DNA polymerases during replication, DNA repair enzymes, and proteins involved in recombination and cell cycle [1]. The Candida Genome Database annotates *C. albicans* orf19.4616 as yeast PCNA homologue [37] and mRNA expression profiling study during cell cycle suggested overexpression of this ORF during G1/S transition along with other proteins involved in DNA synthesis [39]. Separation of proteins from *Candida* cells by 2-D gel electrophoresis revealed an increased amount of CaPol30 when cells were growing exponentially than the stationary phase cells [40]. Similarly, genome wide expression profiling in response to fungal drugs like 5-FC suggested increased expression of POL30 [41]. Tyrosol, a quorum-sensing molecule in *C. albicans* has also been found to regulate the expression PCNA [42]. Despite these facts, PCNA from *C. albicans* had not been characterized until this study. In this report for the first time we have presented an extensive study on cloning and characterization of CaPCNA; and reported functional similarities and differences between CaPCNA and ScPCNA.

Our study suggests that orf19.4616 encodes for a classical eukaryotic DNA clamp. Primary sequence and molecular modeling analyses suggest a great degree of conservation in various motifs and the three dimensional structures among PCNAs. Like other eukaryotic PCNAs, CaPCNA also forms a ring of three monomers; each consisting of two topologically similar globular domains connected by a hydrophobic loop. The folding of inter domain connecting loop of CaPCNA is also undistinguishable from that of ScPCNA, as IDCL is known to bind to DNA polymerases, accordingly a conserved interaction with yeast DNA polymerase eta was also established. For the first time, we designed a urea-PAGE based study to check the stability of PCNA ring and found that both candida and yeast PCNA were equally resistant to urea dependent denaturation. Glutaraldehyde cross linking experiment also revealed formation of double trimers as has been shown in the case of human PCNA [34]. Although the exact role of double trimeric PCNA ring has not been established, its physiological advantage of interacting with multiple partners at a given time and process can be speculated.

Remarkable structural conservation and homology in primary sequences of CaPCNA resulted in complementation of essential function of yeast PCNA deletion mutant. Similar observation has been reported for human PCNA supporting viability of a *Schizosaccharomyces pombe* deletion mutant [21]. The PCNA of *Drosophila melanogaster* but of *S. cerevisiae* facilitates *in vitro* DNA replication of SV40 [43]. This suggests existence of similar interactions between PCNA and replicative DNA polymerases; and probably the binding sites are also conserved in eukaryotes. Simultaneously, it also implies that ScRFC, the clamp loader cannot differentiate CaPCNA against ScPCNA while loading. This finding is well supported by an observation that the PCNA-partner interaction networks are coevolved in *S. cerevisiae* and *C. albicans* due to their highly conserved interaction regions in both PCNA and interacting proteins. Therefore PCNAs of these two fugal species are in the same compatibility group distinguished from that of *Y. lipolytica, A. nidulans, N. crassa,* and *S. pombe* [44]*.* The comparatively slow growth phenotype, susceptibility to non-permissive temperatures and sensitivity to HU of CaPCNA containing yeast strain could be due to absence of some of the critical contacts between CaPCNA and yeast replicative polymerases like Polδ or Polε [3]; and thereby although supporting DNA replication, but with reduced efficiency. In fact, DmPCNA also supports *in vitro* SV40 DNA replication with compromised efficiency [43]. However, optimal growth was observed in the wild type *Candida* strains SC5314 and DKCa39; and even better growth rate in comparison to wild type yeast strains suggesting a species-specific interaction between PCNA and its partner is beneficial during DNA replication. Biochemical studies suggested G178S mutants of *Candida* and yeast PCNA behave similarly. However, CaPCNA G178S mutant did not complement yeast for its deficiency of ScPCNA whereas the corresponding yeast PCNA mutant did. Also the *pol30-178* of *S. cerevisiae* allelic strain that carries a genomic mutation of G178 to serine mutation in PCNA survives and did not show any replication defects [32]. Another *pcna-52* allele which carries S115P mutation and the corresponding purified proteins remaining as monomers during gel filtration and even multimers by crosslinking experiments with glutaraldehyde have also been reported [18]. Although these mutant proteins abundantly form monomers and some population of multimers *in vitro*, apparently in the cell the PCNA trimeric rings get stabilized by some yet to be identified mechanism(s).

An array of site directed and random mutants of ScPCNA have been extensively studied to understand its differential role in DNA replication and repair processes [18, 19]. Most of the mutations were on the outer surface of the ring required for protein-protein interactions and exhibited wide range of sensitivity to MMS, UV and HU. One class of mutants like *pcna-6* (DE41,42AA), *pol30-45* (RDDE61,63,256,257AAAA), *pol30-46* (EDDE104,105,256,257AAAA), *pcna-79* (IL126,128AA) and *pcna-90* (PK252,253AA) were susceptible to hydroxyurea, exhibited compromised growth and DNA repair defects. A number of other PCNA mutants have also been characterized those are defective in replication also show defects in DNA repair. Whereas second class of mutants like *pol30-9* (ED104,105AA), *pol30-22* (DE256,257AA), *pcna-201* (C22Y) and *pcna-204* (C81R) alleles did not show any phenotypes linked to DNA replication defects but showed DNA repair defects. Particularly, *pcna-201* (C22Y) and *pcna-204* (C81R) alleles are defective in mismatch repair. Limitation of these analyses is that most of the PCNA that showed defects in replication and repair carry multiple mutations that may alter the structure and property of PCNA. As biochemical characterization of some of these mutant PCNAs like PCNA-240, PCNA-45 showed formation of monomeric PCNA and compromised interaction with DNA repair related proteins [19]; thus it may be argued against the suggestion of separate role of PCNA in replication and repair. Therefore similar studies with a naturally occurring eukaryotic PCNA will be a novel approach to delineate the precise roles of PCNA in DNA replication or in repair. In this study not only we have first time reported characterization of CaPCNA, but also we have presented a naturally occurring PCNA form *C. albicans* whose mere presence in yeast showed characteristics of replication defects like slow growth, smaller colony size, temperature susceptibility and sensitivity to HU; and DNA repair defects like sensitivity to DNA damages with agents like MMS, H_2_O_2_ and TBHP. It was unexpected as interestingly, PCNA2 from *A. thaliana* plant not only supported viability of *pol30Δ S. cerevisiae* strain, it also conferred resistance to DNA damaging agents [45]. Nevertheless, CaPCNA belongs to a class of ScPCNA mutants like *pcna-6, −79, −90* and others that show both DNA replication and repair defects but support cell viability.

## Conclusions

We conclude that orf19.4616 encodes for a classical eukaryotic DNA clamp and shares high degree of similarities in its biochemical and structural features with ScPCNA. Although both *S. cerevisiae* and *C. albicans* belong to same fungal family of Saccharomycetaceae and despite both the PCNAs possessing common features, CaPCNA is not fully compatible in replacing ScPCNA in processes like DNA replication and repair in yeast. Remarkable tolerance to DNA damaging agents phenotype exhibited by *C. albicans* strains compared to yeast strains suggest that *C. albicans* being an opportunistic pathogen genetically evolved to tolerate extreme conditions and probably to escape from attack by macrophages to maintain its genomic blue prints. Unlike *S. cerevisiae*, as BER, NER, MMR and NHEJ pathways have been ruled out to play a major role in genomic stability in *C. albicans* [29, 30], PCNA coordinating other DNA damage tolerance pathways such as replication, translesion DNA synthesis and recombination could be playing an important role that requires further investigation. Considering all the evidences, we conclude that despite structural and physiochemical similarities; there are distinct functional differences between ScPCNA and CaPCNA, and the ways both the strains maintain their genome stability. Hence PCNA may play a critical role in the development of systemic candidiasis and targeting PCNA could develop a new means to combat *C. albicans* infection.

## Materials and Methods:

### Oligonucleotides, strains and media

The oligonucleotides used in this study were procured from Integrated DNA Technologies (IDT, USA). *S. cerevisiae* EMY74.7 and *Candida albicans* SC5314 were used as wild type strains [37]. A protease deficient yeast strain YRP654 was used for protein expression of ScPolη [46]. For plasmid segregation analysis, YTS9 which is a *POL30* genomic null yeast strain supported by a YCplac22 (CEN, TRP) plasmid derived *POL30* with DD41,42AA mutations was used. Various yeast strains obtained by transforming different PCNA constructs to YTS9 and others used in this study are described in Table 1. *C. albicans* and *S. cerevisiae* strains were grown on YPD media with or without DNA damaging agents or on various synthetic drop out media as required.

**Table 1:** Strains used in this study

Strain	Genotype	Source/ Reference
SC5314	*C. albicans* Wild type	[25]
EMY74.7	*MATa, his3-Δ1, leu2-3, leu2-112, trp1Δ, ura3-52*	[32]
HFY7C	*MATa, ura3-52, his3-200, ade2-101, lys2-80,1 trp1-901,*	
	*leu2-3,112 gal4-542, gal80-538, LYS2::GAL1-HIS3*	Clontech
	*URA3::(GAL4 17mer)*_*3*_ *-CYC1 lacZ*	
YTS9	*MATa ura3-52 trp1D901 leu2-3,112 can1 pol30Δ1,* with pBL230-6 (YCP50, TRP1, POL30 with DD41,42AA)	Ayyagari etal
YNA01	YTS9 with pNA1005 (2 μ, ADH1p,URA3)	This study
YNA03	YTS9 with pNA1006 (2 μ, ADH1p-CaPCNA,URA3)	This study
YNA05	YTS9 with pNA1012 (2 μ, ADH1p-ScPCNA,URA3)	This study
YNA07	YTS9 with pNA1005(2 μ, ADH1p-Ca PCNA G178S,URA3)	This study
YNA09	*MATa ura3-52 trp1D901 leu2-3,112 can1 pol30Δ1,* pNA1006	This study
YNA11	*MATa ura3-52 trp1D901 leu2-3,112 can1 pol30Δ1,* pNA1012	This study
YNA13	YTS9 with pNA901(2 μ, CaPCNA gene,URA3)	This study
YNA15	YTS9 with pNA903(2 μ,CaPCNA gene G178S,URA3)	This study
YNA21	YTS9 with YCplac33	This study
YNA23	YTS9 with pNA983(CEN, CaPCNAgene, URA3)	This study
YNA25	YTS9 with pNA984(CEN,URA3,CaPCNAgene G178S)	This study
YNA27	YNA11 with YEp lac181	This study
YNA29	YNA11 with pNA1186 (2 μ, ADH1p-Ca PCNA, LEU)	This study
YNA31	YNA11 with pNA1188 (2 μ, ADH1p-Ca PCNA G178S, LEU)	This study
YNA33	YNA11 with pNA1194 (2 μ, CaPCNA gene LEU)	This study
YNA35	YNA11 with pNA1209 (2 μ, ScPCNA, LEU)	This study
YNA41	*MATa ura3-52 trp1D901 leu2-3,112 can1 pol30Δ1,* pNA901	This study
YNA43	*MATa ura3-52 trp1D901 leu2-3,112 can1 pol30Δ1,* pNA983	This study
YNA45	YNA11 with pNA1209 (2 μ, ScPCNA G178S, LEU)	This study

### Generation of PCNA constructs

A 50 μl PCR reaction was carried out containing 100 ng of SC5314 genomic DNA, 250 μM dNTPs, 1 × Pfx enzyme buffer, 1.5 mM Mg_2_SO_4_ and 2U of Pfx DNA polymerase (Invitrogen) and 20pmol each of the primers NAP31 (5’-GGC CAA GCT TGG ATC CAC ATA TGT TAG AAG GTA AAT TTG AAG -3’) and NAP32 (5’- GGC CGA ATT CGG ATC CCT ACT CAT CAT CAT CG-3’) for the orf 19.4616; and NAP29 (5’- CCG GAA GCT TAC GAT GCT TAT AGA TTG-3’) and NAP30 (5’-GGC CGA ATT CGA AGA GTA GTT AAC ATT TG-3’) for the gene, respectively. PCR conditions included initial heating at 95 °C for 3’ followed by 34 cycles at 95 °C for 30s, 48 °C for 45 s, 68 °C for 50s (orf) or 1’ 30s (gene). Amplified PCR products (780 and 1449 bp) were purified, digested with HindIII-EcoRI, and cloned into the same sites of pUC19. Further the clones were authenticated by sequencing. The pathogenic yeast *C. albicans* decodes CUG codon as serine instead of leucine, however CaPCNA does not harbour any such codons in its orf; and therefore no further modification was required for expression [37, 47]. The BamHI fragment containing wild type or G178S PCNA orf was further subcloned into BglII site of a bacterial expressed amino terminal GST-tag construct. Similar fragments were also subcloned into BamHI site of an *ADH1* promoter based yeast expression system. The PCNA gene was also subcloned to yeast vectors (YEp/YCp) for complementation analysis.

### Site directed mutagenesis

G178S mutation was generated on respective pUC19 constructs by an inverse PCR approach using *Pfx* DNA polymerase with primer pairs NAP218 (5’- GAT TCT GGT TCC TCA AGT GTT ATC TTG-3’) and NAP219 (5’-CAA GAT AAC ACT TGA GGA ACC AGA ATC-3’) for CaPCNA; and NAP284 (5’-CGG TGA CAT CGG ATC ATC ATC AGT CAT AAT AAA ACC-3’) and NAP285 (5’-GGT TTT ATT ATG ACT GAT GAT GAT CCG ATG TCA CCG-3’) for ScPCNA, respectively. PCR conditions included 1 cycle of 95 °C for 3 min, 20 cycles of 95 °C for 1 min, 50 °C for 30 s, and 68 °C for 3.5 min, and finally 1 cycle of 68 °C for 10 min. The PCR product was treated with DpnI (NEB) overnight and transformed into *E.coli* TG1 competent cells. The colonies were screened by sequencing.

### Purification of recombinant proteins

The expression of PCNA proteins were carried out in *E. coli* BL21-DE3 strain. The proteins were expressed as glutathione S-transferase fusion proteins under T7 promoter by using a protocol described previously [46, 48]. Briefly, 10 ml preculture was inoculated into 1lt LB + 100 μg/ml ampicillin and grown at 37 °C to an OD_600_ of 0.6. Further the culture was induced by adding 1 mM IPTG, and incubated for an additional 6 to 8 h. About 5gm of cell pellet was resuspended in 20 ml cell breaking buffer (50 mM Tris–HCl pH 7.5, 10 % sucrose, 1 mM EDTA and 500 mM NaCl) containing a 1:200 dilution of the protease inhibitor mixture, suspension was pressure lysed using a French press at 20,000 psi at 4 °C. The lysate was clarified by centrifugation at 10,000 RPM for 10 min, and further at 20,000 RPM for 1 h. The clear supernatant was passed twice through GST beads (GE-healthcare) packed in 10 ml plastic column. Protein bound beads were first thoroughly washed using the above mentioned buffer with 1 M NaCl, and then with equilibration buffer (50 mM Tris–HCl pH 7.5, 150 mM NaCl, 10 % glycerol, 5 mM DTT and 0.01%NP-40). The protein was eluted from the beads after overnight incubation with Prescission protease (GE-Healthcare) that cleaves PCNA from GST. Purified proteins were estimated by both Bradford’s and gel based assay using bovine serum albumin as a standard. Proteins were analyzed by electrophoresis on 12 % polyacrylamide gels without or containing either 0.1 % SDS or 2–6 M urea [49], and visualized by Coomassie Brilliant Blue R-250 staining .

ScPolη was also purified by above mentioned protocol except that GAL-PGK-GST-ScRAD30 construct was expressed in YRP654 protease deficient yeast strain [48].

### Glutaraldehyde cross-linking

About 20 μg of native or mutant PCNA in 20 mM HEPES buffer (pH 7.5) was mixed with 1 μl of 8 % freshly prepared glutaraldehyde solution for 5 min at 37 °C. The reaction was terminated by adding equal volume of 1 M Tris–HCl (pH 8.0). Cross-linked protein was solubilized with sample buffer and electrophoresis was conducted on 12 % SDS-PAGE.

### Size exclusion chromatography

For size exclusion chromatography, about 10 μg of purified proteins were loaded onto a Superdex 200 PC3.2/30 column pre-equilibrated with buffer containing 50 mM HEPES pH7.5, 150 mM NaCl and 10 % glycerol. Chromatography was performed on an AKTApure M system (GE Healthcare) at a flow rate of 0.05 ml/min at 4 °C, and the absorbance was monitored at 280 nm.

### Complementation analysis by plasmid curing

Plasmids YCplac33 (CEN, URA3) or YEplac195 (2 μ, URA3) harbouring *CaPOL30* or *CaPOL30 G178S* genes, and YEplac195-ScADH1p (2 μ, URA3) expressing CaPol30 or CaPol30 G178S or ScPol30 orfs were transformed into YTS9 strain and the transformants were selected on synthetic media without tryptophan and uracil. For control experiments empty vectors were also transformed. In order to cure the resident plasmid containing mutant *ScPOL30* (YCP-*ScPOL30 DE41,42AA*, TRP1), a transformant from each plate was grown on liquid media omitting uracil at 30 °C in shaking condition. After every 24 hrs, 100 μl of the culture was diluted with fresh 5 ml of SD w/o uracil media and allowed to grow. After seven such consecutive sub-culturing, the cultures were streaked on SD w/o uracil plates to obtain isolated colonies. About 30 isolated colonies were patched on both SD agar plates lacing uracil alone or tryptophan alone. Further the plates were incubated at 30 °C for 2 days and number of colonies appeared on each plates were counted. The lack of growth on SD-tryptophan plate but growth on SD-uracil suggested complete curing of the resident PCNA and complementation due to the incoming plasmid. On the other hand, preservation of both the nutritional markers (URA and TRP) indicated that the tester plasmid is unable to complement for Sc*POL30*. After curing, the strains were confirmed by PCR for the presence of respective plasmids.

### Plasmid segregation by 5-FOA counter selection

Yeast strain YNA05 is a genomic null for *POL30* but harbors YEplac195-ScADH1p-ScPol30 (URA3). An empty YEplac181-ScADH1p (LEU2) or containing CaPol30 or CaPol30 G178S or ScPol30 G178S orfs were transformed into above strain. *Candida* PCNA with its own promoter in YEplac181 vector was also transformed. The transformants were streaked on SD media w/o leucine and uracil. Isolated colonies were picked and re-streaked on SD-leucine agar plate but with or without 5′-fluoroorotic acid (10 mg/ml). After counter selection with 5′-FOA, the absence of original PCNA in strains surviving on FOA containing plate was confirmed by PCR.

### Growth rates

Growth rates for yeast strains were examined by inoculating the required volume of pre-culture to 100 ml fresh YPD liquid medium to get 0.1 O.D_600_ at 0 h. About 1 ml culture was taken out in every 1 h for 18 hrs and the growth rate at 30 °C was monitored by measuring absorbance at 600 nm. Growth of the strains was also monitored at 14 °C and 35 °C.

### RT-PCR analysis

Total RNAs were isolated from various yeast stains those were used in estimating growth rates by using MP FastRNA Pro isolation kit (Cat#6025-050) as per the manufacturer’s protocol and then samples were treated with DNAase I enzyme. Equal amount of RNAs were subjected to cDNA synthesis using a reverse transcription core kit from Eurogentec (RT-RTCK-03). A 50 μl PCR reaction was carried out containing 3 μl of synthesized c-DNA, 200 μM dNTPs, 1 × Taq DNA polymerase buffer, 1.5 mM MgCl_2_ and 2U of Taq DNA polymerase (Sigma) and 20pmol each of the primers NAP31 (5’-GGC CAA GCT TGG ATC CAC ATA TGT TAG AAG GTA AAT TTG AAG -3’) and NAP32 (5’- GGC CGA ATT CGG ATC CCT ACT CAT CAT CAT CG-3’) for the CaPCNA; and NAP205 (5’- GGC CGG TAC CGG ATC CAT ACA TGT TAG AAG CAA AAT TTG-3’) and NAP210 (5’- GGC CAA GCT TGG ATC CTT ATT CTT CGT CAT TAA ATT TAG G-3’) for the ScPCNA, respectively. Primers pair NAP297 (5’-GGC CGG ATC CGT ATG TCA AAA TTT ACT TGG -3’) and NAP298 (5’-GGC CGG ATC CTC ATT TTT TTC TTG TAA AAA ATG-3’) were used for amplification of ScPolη. PCR conditions included initial heating at 95 °C for 3’ followed by 25 cycles at 95 °C for 30s, 52 °C for 45 s, 68 °C for 1’ (PCNA) or 1’ 45 s (Polη). PCR products were separated on 1 % agarose gel and image captured on Chemi XRS Gel Documentation System (Bio-Rad). PCR reaction was also repeated with RNA alone as template to rule out any genomic DNA contamination in our preparation. Intensity of each band was calculated by ImageJ software and relative fold change of PCNA vs Polη was estimated.

### Sensitivity to different DNA damaging agents

All the pre-cultures of various strains were diluted to achieve equal number of cells in autoclaved water. Further these strains were 10 folds serially diluted and spotted on YPD plates containing with or without different concentrations of drugs like hydroxyurea (HU), methyl methane sulfonate (MMS), H_2_O_2_ and *tert*-Butyl hydroperoxide (TBHP). The plates were incubated at 30 °C for 3 days and then photographed. Uridine (100 μg/ml) was added when required.

### Physical interaction by Surface Plasmon Resonance

Interaction of PCNA with DNA polymerase eta (Rad30/η) was monitored using Bio-Rad XPR 36 surface plasmon resonance biosensor instrument. About 5 μg of CaPCNA or ScPCNA or BSA (~350 RU) was immobilized on GLC chip by amine coupling method as suggested by manufacturer’s instructions. ScPolη was injected at concentration ranging from 25-500nM with running buffer composed of 25 mM HEPES pH 7.5, 10 % glycerol, 200 mM Sodium acetate pH 7.8, 8 mM Magnesium acetate, 1 mM DTT, 0.005 % Tween-20 and 0.2 mg/ml BSA, at a flow rate of 50 μl/min for 180 s with a 600 s dissociation phase. Molecular interaction was carried out at 20 °C. Further the dissociation constants were determined, after fitting the association and dissociation curves to a 1:1 (Langmuir)-binding model.

### Bioinformatics analysis of Candida PCNA

The BLAST server from the NCBI was used to search for similarities between *Candida* PCNA protein and sequences deposited in the Protein Databank (PDB). Based on the structures of homologues proteins, CaPCNA was modelled using SWISS MODEL (http://swissmodel.expasy.org/) [50], an automated protein modelling Server. *Saccharomyces cerevisiae* PCNA PDB structures with ID 2OD8 and 1PLR were used as template for monomeric and trimeric PCNA, respectively. Stereochemical quality of the model structured of CaPCNA was further analysed by the metaserver SAVES. The Ramachandran plot was derived by PROCHECK and secondary structure prediction of CaPCNA was performed with PSIPRED v3.3 softwares [51, 52].

## Additional files

Additional file 1:
**Table S1.** Average O.D. _600_of three sets of experiments (XLSX 11 kb) Additional file 2:
**Table S2.** Quantification of band intensities of Semi-quantitative RT PCR for yeast expressing various PCNAs (XLSX 11 kb)

## Abbreviations

PCNA: Proliferating Cell Nuclear Antigen
IDCL: Inter Domain Connecting Loop
RFC: Replication factor C
MMR: Mismatch Repair
NHEJ: Non-homologous End Joining
ADH: Alcohol Dehydrogenase
5-FOA: 5-Fluoroorotic Acid
GST: Glutathione S-trasferase
BSA: Bovine Serum Albumin
RU: Response Unit
RMSD: Root mean square deviation
Polη: DNA polymerase eta
HU: Hydroxyurea
UV: Ultra Violet
MMS: Methyl Methanesulfonate
H_2_O_2_: Hydrogen Peroxide
TBHP: tert-Butyl Hydroperoxide
CEN: Centromere
ROS: Reactive Oxygen Species
RNI: Reactive Nitrogen Intermediates
